# Apophyseal injuries in adolescent athletes: the multimodal radiological perspective

**DOI:** 10.1590/0100-3984.2025.0059ed

**Published:** 2026-09-08

**Authors:** Leonor Garbin Savarese

**Affiliations:** 1 Department of Medical Imaging, Hematology, and Clinical Oncology, Ribeirão Preto Medical School, University of São Paulo, Ribeirão Preto, SP, Brazil. E-mail: lsavarese@hcrp.usp.br

The growing participation of children and ado-lescents in competitive sports has introduced new diagnostic challenges to musculoskeletal radiology. Among these, apophyseal injuries in adolescent soccer players stand out. In young athletes with an immature skeleton, the physeal plate is two to five times weaker than the surrounding fibrous structures and therefore more vulnerable to injury^**(^1^)**^. Differentiating between apophyseal avulsion, typically acute and abrupt in onset, and apophysitis, which results from chronic microtrauma, is crucial for appropriate clinical man-agement, prevention of recurrence, and determination of the time to return to play. In this context, imaging evaluation plays a decisive role, not only in diagnosis but also in therapeutic guidance^**(^2^,^3^)**^.

In this scenario, the article by Mogami et al.^**(^4^)**^, published in **Radiologia Brasileira**, represents a valuable contribution by integrating three imaging modalities-plain radiography, ultrasonography with power Doppler, and magnetic resonance imaging (MRI)-in the assessment of apophyseal avulsions in adolescent soccer players. The authors system-atically quantified the diagnostic performance of each method and proposed practical correlations among their findings.

The authors demonstrated that plain radiography, although widely available and characterized by high specificity and positive predictive value, has limited sensitivity, being useful to confirm but not to exclude avulsions. Power Doppler imaging findings showed a significant association with avulsions detected on MRI, underscoring the potential of power Doppler as a complementary tool for identifying post-traumatic neovascularization, an imaging marker of tissue repair that remains underexplored in sports radiology. MRI, in turn, allowed the distinction between character-istic patterns of avulsions (edema surrounding the apophysis and intermuscular fluid collections) and apophysitis (diffuse bone marrow edema), expanding the understanding of the spectrum of these lesions. Despite inherent limitations related to sample size, the cross-sectional nature of the study, and the unequal distribution of imaging modalities, the results are consistent and support the relevance of an integrated diagnostic approach.

Although MRI remains the gold standard, the integration of radiography and ultrasonography with power Doppler appears promising as an initial diag-nostic strategy, particularly in resource-limited set-tings. In the study in question^**(^4^)**^, all athletes who exhibited hyperemia on Doppler imaging had avulsions confirmed on MRI, whereas no cases of apophysitis or normal MRI showed abnormal vascular flow, an observation of high specificity. Conversely, negative Doppler findings did not exclude avulsion, given that they were also observed in confirmed cases. These results highlight the potential of power Doppler ul-trasonography as a complementary triage tool, espe-cially in sports environments that require rapid and cost-effective decision-making.

The implications of this work are broad and promising and should encourage further research into the role of imaging techniques, particularly ultrasonography, in the evaluation of young athletes with apophyseal pain. Future studies could explore the performance of B-mode ultrasonography alone or in combination with radiography. If this combination proves sufficiently accurate for initial assessment, it might represent a practical, cost-effective alternative in settings where MRI is not readily available. The article by Mogami et al.^**(^4^)**^ stands out for expanding the body of knowledge on the spectrum of apophyseal injuries and for paving the way toward a multimodal approach that is more integrated and efficient in the evaluation of adolescent athletes.
